# Management Strategies for Sexuality Complaints after Gynecologic Cancer: A Systematic Review

**DOI:** 10.1055/s-0042-1756312

**Published:** 2022-09-29

**Authors:** Luciane Machado Pizetta, Augusto da Cunha Reis, Mirian Picinini Méxas, Vanessa de Almeida Guimarães, Carmen Lucia de Paula

**Affiliations:** 1 Centro Federal de Educação Tecnológica Celso Suckow da Fonseca, Rio de Janeiro, RJ, Brazil; 2 Universidade Federal Fluminense, Niterói, RJ, Brazil

**Keywords:** survivors, gynecological cancer, sexuality, supportive care needs, sobreviventes, câncer ginecológico, sexualidade, suporte de necessidades de cuidados

## Abstract

**Objective** To explore the main sexuality complaints of gynecologic cancer survivors after treatment and to identify the care strategies provided.

**Data Source** Searches were conducted in six electronic databases: Scopus, Web of Science, LILACS, MEDLINE, PsychINFO, and EMBASE.

**Study Selection** Articles published between 2010 and 2020 were selected and the following descriptors were used in the English language: *female genital neoplasms* and *gynaecological cancer* . The methodological quality of the studies used the Mixed Methods Appraisal Tool (MMAT).

**Data Collection** The primary data extracted were: names of the authors, year of publication, country of origin, objective and type of study, data collection instrument, sample size and age range, types of cancer, and symptoms affected with the strategies adopted.

**Data Summary** A total of 34 out of 2,536 screened articles were included. The main strategies found for patient care were patient-clinician communication, practices for sexuality care, individualized care plan, multiprofessional team support, and development of rehabilitation programs. For sexuality care, the most common practices are pelvic physiotherapy sessions and the use of vaginal gels and moisturizers.

**Conclusion** The main complaints identified in the scientific literature were low libido and lack of interest in sexual activity, vaginal dryness, pain during sexual intercourse, and stenosis. Different care strategies may be adopted, such as follow-up with a multidisciplinary health team and sexual health rehabilitation programs, which could minimize these symptoms and ensure the quality of life of patients.

## Resumo

**Objetivo** Explorar as principais queixas da sexualidade com sobreviventes de câncer ginecológico após o tratamento e identificar as estratégias de cuidados prestados.

**Fonte dos Dados** As buscas foram realizadas em seis bases eletrônicas: Scopus, Web of Science, LILACS, MEDLINE, PsychINFO e EMBASE.

**Seleção dos Estudos** Foram selecionados artigos publicados entre 2010 e 2020 e os descritores utilizados (em inglês) foram *female genital neoplasms* e *gynaecological cancer* . A qualidade metodológica dos estudos utilizou a ferramenta Mixed Methods Appraisal Tool (MMAT).

**Coleta de Dados** Os principais dados extraídos foram: nomes dos autores, ano de publicação, país de origem, objetivo e tipo de estudo, instrumento para coleta de dados, tamanho da amostra e faixa etária, tipos de câncer, os sintomas acometidos e as estratégias adotadas.

**Síntese dos Dados** Dos 2,536 artigos identificados, 34 foram incluídos. As principais estratégias encontradas para os cuidados aos pacientes foram a comunicação paciente-médico, práticas para os cuidados sexuais, plano de cuidados individualizado, apoio a equipes multiprofissionais e desenvolvimento de programas de reabilitação. Para os cuidados de sexualidade, as práticas mais comuns são sessões de fisioterapia pélvica e o uso de géis vaginais e hidratantes.

**Conclusão** As principais queixas identificadas na literatura científica foram baixa libido e falta de interesse na atividade sexual, secura vaginal, dor durante a relação sexual e estenose. Diferentes estratégias de cuidados podem ser adotadas, como o acompanhamento com uma equipe de saúde multidisciplinar e programas de reabilitação da saúde sexual, as quais poderiam minimizar estes sintomas e garantir a qualidade de vida dos pacientes.

## Introduction

Early detection and treatment of gynecologic cancer at an initial stage allow high chances of cure and improved survival and, consequently, an increase in the number of survivors. 1 2 3 4 5 6 7 8

All forms of treatment for gynecologic neoplasia (pelvic surgery, radiation, and drug therapy) have the potential to impair physiological and psychological functions, and also generally affect the self-esteem, body image, femininity, and intimate relationships of women; sexual dysfunction is the most frequently mentioned in the literature. 9 10 11 12

Sexual health is an essential aspect of quality of life. As a result of the aggressive treatment received, > 40% of gynecologic cancer patients reported chronic and distressing sexual difficulties, even after 12 months of treatment. 13 14 15 Sexual dysfunction, in addition to being extremely distressing, negatively affects the relationship with their spouses, which can result in emotional estrangement. 16 17 18 19

Studies indicate that 74% of the surviving participants felt that communication with oncology professionals about sexual issues was important, but few had received this information. 20 21 Many women reported having no discussion about sexuality with their physicians during and after treatment, which represents a troubling gap in the provision of supportive care for health needs. 22 23

Recognizing that post-treatment gynecologic cancer causes various negative impacts on the health of the female sexual organs, it becomes important to seek to understand the subjective phenomena about the experience of sexuality after treatment. 24 25 Recently, topics such as “cancer survivors” and “supporting care needs” have been increasingly published, and many researchers are studying these concepts, addressing dimensions such as “sexuality”, “gynecologic cancer”, and “health dysfunctions after cancer.” 26 27 28 29 30

Various care strategies that can help survivor patients are mentioned in the scientific literature, such as the development of support groups with the purpose of providing an environment of cooperation, solidarity, and readaptation to the disease, multiprofessional care team support to minimize the difficulties associated with the disease, doctor-patient communication, and development of rehabilitation programs. 31 32 33 34

However, there are strategies that may present disadvantages such as the deficiency of clinical training by health professionals to perform an appropriate approach, because many patients have personal difficulties and taboos about sexuality. 35 36

About 65% of gynecologic cancer patients do not have their needs met; little is explored about knowledge on the experience of supportive care for gynecologic cancer survivors or the supportive resources available. 37

In this regard, there is a need for a literature review to investigate new resources that can be developed and incorporated into treatment and healthcare settings for the benefit of patients.

The current review aimed to explore the main collateral effects on sexuality with gynecologic cancer survivors after treatment and to identify the care strategies provided.

## Methods

### Search Selection Strategy

For the development of the present study, a systematic literature review was performed based on the Preferred Reporting Items for Systematic Reviews and Meta-Analyses Protocol (PRISMA-P). 38 The search for articles was performed in the following electronic databases: Scopus, Web of Science, LILACS, MEDLINE, PsychINFO, and EMBASE. Searches in the electronic databases was performed from January to February 2021. The search strategies were changed according to the requirements of each database. Initially, two authors chose independently the articles selected for eligibility, following a two-phase process. The initial screening phase resulted in a restricted list of articles, including titles and abstracts. In the second stage, the screening process involved reading the articles in full text. The two reviewers independently assessed all articles for eligibility concerning the selection criteria until consensus was reached. When there was disagreement and no consensus was reached, a third author reviewed the articles and selected the one with the greatest adherence to the research. The qualitative synthesis of the selected papers was based on an integral lecture focusing on post-treatment gynecological neoplasms ( Chart 1 ).

**Chart 1:** Search terms and strategy

Search ID#	Search terms	Search mode
S1	*female genital neoplasm* OR *uterine neoplasm* OR *vulvar neoplasm* OR *vaginal neoplasm*	Boolean/Phrase
S2	*ovarian neoplasm* OR *female genital cancer* OR *uterine cancer* OR *vulvar cancer* OR *vaginal cancer*	Boolean/Phrase
S3	*ovarian cancer* OR *female genital tumors* OR *uterine tumors* OR *vulvar tumors* OR *vaginal tumors*	Boolean/Phrase
S4	*ovarian tumors* OR *gynaecological cancer* OR *gynecological cancer* OR *gynaecological tumors* OR *gynecological tumors*	Boolean/Phrase
S5	*female genital carcinoma* OR *uterine carcinoma* OR *vulvar carcinoma* OR *vaginal carcinoma* OR *ovarian carcinoma*	Boolean/Phrase
S6	*sexual health* OR *sexuality* OR *sexual function*	Boolean/Phrase
S7	*patient-centered care* OR *patient care* OR *supportive care* OR supportive care need* OR *helping behavior* OR *humanization* OR *survivorship* OR *survival* o OR r *cancer survivorship*	Boolean/Phrase

### Inclusion and Exclusion Criteria

To be considered eligible, the following inclusion and exclusion criteria was applied to all identified scientific articles.

### Inclusion Criteria

- Studies that investigate care support needs of women survivors of gynecological cancer (post-treatment of the disease) with a focus on sexuality.
- Studies employing quantitative and/or qualitative methods, regardless of research design.
- Studies published in English with readily available abstracts.
- Studies performed with adult individuals ≥ 18 years old diagnosed with gynecological cancer (endometrial, cervical, ovarian, and vulvar) including the effects and aftertreatment mainly affecting sexuality.
- Articles focusing on the medium and long-term effects of gynecological cancer.
- Studies published as original articles in specialized journals between 2010 and 2020.

### Exclusion Criteria

- Studies in which care support needs of survivors of gynecological cancer focused on sexuality were not explicitly discussed.
- Studies conducted with patients with other types of cancer, whether primary or secondary.
- Systematic reviews and individual publications within systematic reviews were excluded so as not to duplicate the content.

### Data Extraction and Analysis

Data from the included studies were extracted into a predefined data extraction table. Data extraction was performed by a reviewer and then checked by a second reviewer jointly and according to the agreement and consistency between them. A third reviewer analyzed discrepancies. Three time periods were established:

1. Immediately after treatment (0 to 3 months post-treatment).
2. Long-term post-treatment effects (> 3 months to 5 years).
3. Mixed (Studies with patients from both time frames, that is, from 0 to 3 months to 5 years post-treatment.

The included studies were classified according to the type of studies conducted and to the location of the tumor according to discussions with the research team. The quality of the studies was independently assessed by two reviewers using the Mixed Methods Appraisal Tool (MMAT).

### Risk of Bias

The articles were independently assessed to maintain methodological quality. The negative and positive points were analyzed and, at the end, a score from 0 to 4 was attributed to each article (0 = no criteria fulfilled, 1 = fulfilled one criterion, 2 = fulfilled two criteria, 3 = fulfilled three criteria, and 4 = fulfilled all four criteria). 39 When any disagreement occurred, the authors discussed them to reach a final agreement.

### Qualitative Synthesis

After the search for studies in the databases, a screening was performed independently by the review team by reading the titles and abstracts. This search in scientific databases generated the return of a large number of articles, and the strategy adopted was to ensure sensitivity on the specificity and compliance to the established criteria. Thus, for the screening of studies, the sum of the total number of articles in all databases was recorded; the titles were read quickly, allowing the selection of references, and discarding those that did not fit the eligibility criteria established by the review team. A free reference management software, Mendeley, was used to sort the articles, count duplicate articles, and organize the references, thus providing greater practicality and time optimization. The studies that passed the screening had their full text retrieved, and the eligibility of studies was confirmed after reading the full text and selection of studies that investigate support care needs of survivors of gynecological cancer (post-treatment of the disease) with a focus on sexuality.

## Results

### Study Selection

**Fig. 1:**
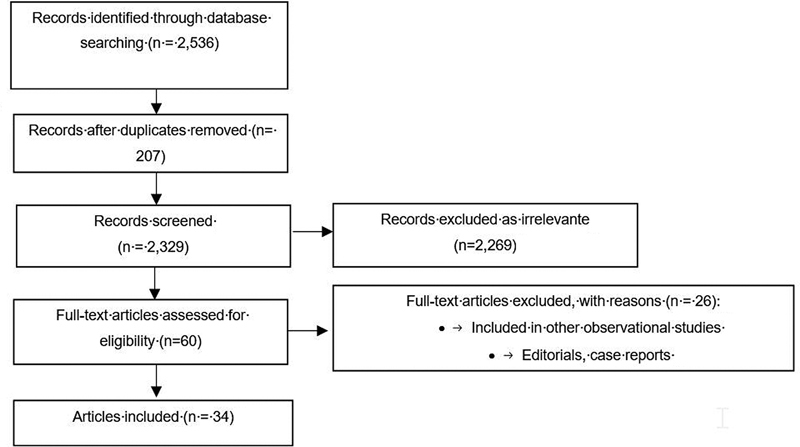
Flowchart of the process of search and selection of studies.

Initially, 2,536 potential articles were identified: 102 through Scopus; 153 through PubMed; 1,062 through EMBASE with a “not PubMed” filter; 760 through Medline; 25 through Lilacs; 51 through a concurrent search of CINAHL and PsycINFO on the EBSCO platform; and 383 through Web of Science. Duplicates were removed and 2,329 remaining articles were evaluated based on title and abstract. Most were discarded because they did not meet the inclusion criteria. Only 60 potentially relevant articles were retained and assessed based on full text, leaving 34 included articles ( Fig. 1 ).

### Study Characteristics

The studies included most frequently women who had been diagnosed with gynecological cancer ( *n* = 19; 56%). Only 1 study on cervical cancer was conducted with young adult women (18 to 39 years old). The remaining studies ( *n* = 5; 12%) reported experiences of gynecological cancer survivors regarding its adverse effects and the care that needs to be received in the context of sexuality. The most common countries where the survey was performed were: USA ( *n* = 7; 21%), followed by Canada ( *n* = 5; 15%) and the Netherlands ( *n* = 4; 12%). Few studies ( *n* = 3; 8.8%) included cancer survivors residing in rural or remote locations. Population studies occurred most frequently at an average of 6 months after the end of treatment ( *n* = 18; 54%). The average sample size was 180 participants (range: 11 to 1,029). Most studies had a cross-sectional design ( *n* = 19; 56%). More than 50% of the included studies ( *n* = 19; 56%) were published in the past 5 years. The included studies were mapped using a descriptive synthesis of the domains of the needs, the study population (for example, type of cancer, age), and the post-treatment of the disease with after primary treatment/early survivorship ranging from 1 month to 3 years. Long-term survivorship after 3 years and mixed phase care when samples are composed of survivors with different periods after treatment ( Appendix 1, Supplementary material ).

### Evaluation of the Methodological Quality of the Studies

All 34 studies selected and included in the systematic review were submitted to a methodological assessment by the MMAT instrument; 13 received a score of 4 because they met all criteria in the quality assessment, 19 received a score of 3 because some topics, such as the interpretation of results, were not sufficiently supported by valid data, 2 articles received a score of 2, and no article received the lowest score of 1.

### Main Side Effects of Sexuality Dominance in Women Survivors

According to the selected articles, low libido was identified in 18 studies and was diagnosed as a major problem for gynecological cancer survivors. Therapeutic modalities such as chemotherapy, radiation therapy, and surgeries performed for the treatment of gynecological malignancies can contribute to this problem in various ways. 40 41 42 43 Sexual dysfunction is the main cause of suffering for the patient after gynecological cancer treatment, and ∼ 70% of survivors experience these problems, such as vaginal dryness, pain during intercourse, and vaginal stenosis. 14 15 16 17 18 44 45 These adverse effects can pose great challenges for survivors in the process of readaptation after the treatment phase. 46 47 48 49 50 51 Pain during intercourse was a predominant symptom in 10 of the selected studies and causes a great impact on the lives of women, as many avoid sex and abandon their sex life due to fear of the pain they may experience during intercourse. 9 15 18 46 Vaginal dryness was prevalent in several studies, and it has been identified through research that 70% of sexually active women experienced this side effect. 47 50 51 Currently, with the help of a variety of symptom management offerings, such as vaginal lubricators and targeted therapies, this discomfort can be alleviated for patients. 30 37 49 Studies have reported difficulties for women in adjusting to their altered sex lives and difficulties with intimacy with their partners. 52 53 As a consequence of these problems, many patients experience abandonment by their husband or partner, who does not understand the new context of female sexuality after treatment. 18 23 26 47 49 51 52 53 54 Menopausal symptoms are another side effect associated with female tract cancer, including vulvar, cervical and ovarian cancer. 41 42 Especially in younger women, this causes a devastating effect that interferes with their body image and psychology. 26 36 Sexual distress, although not a physical problem like vaginal symptoms, interferes directly with the psychological sphere (for example, anxiety or depression). 18 36 50 Many women have a poorer quality of life and unfortunately do not look for psychological intervention, which occasionally increases distress rates. 55 56 57 58 A summary of side effects associated with gynecologic cancer patients who had their sexuality affected after treatment is presented in Table 1 .

**Table 1:** Main sexuality complaints of women survivors after treatment

Sexuality	Survival phase		
	After treatment	Long	Mixed*
Low libido	(13, 23, 26, 37, 45, 51, 58)	(27, 56)	(10, 15, 17, 18, 30, 47, 48, 54, 55)
Vaginal dryness	(17, 23, 26, 37, 43, 50)	(46, 49, 51)	(18, 48, 47, 54, 52)
Disinterest in sexual activity	(15, 23, 26)	(49)	(14, 18, 41, 48, 50, 55, 59)
Pain during intercourse	(9, 15, 58)	(51, 56)	(14, 18, 41, 55, 59)
Lower sexual satisfaction	(17)	(46, 56)	(48, 47, 54, 55, 52)
Vaginal stenosis / shortening	(58)	−	(47, 54, 55, 52)
Menopause symptoms	(10, 26, 36)	(51)	(48)
Sexual distress	(18, 26, 36, 57, 58)	(49, 51)	(48, 54)

Note: Mixed-phase comprehends studies that included participants from different treatment periods after cancer, i.e., it covers both studies just after the end of treatment as well as those that ended a few years ago.

### Care and Medical Support Strategies for Collateral Effects on Sexuality

Six strategies were selected to manage adverse effects on sexuality after treatment:

1. Patient-clinician communication
Healthcare providers should understand the sexual, physical, and psychosocial needs of survivors because after treatment the psychological distress of cancer patients is already high, and many professionals overestimate this psychological suffering or fail to detect it, which can lead to detrimental results. 13 47 57 Open sexual communication is an opportunity for patients to seek information and emotional support to adapt positively to sexual changes, as they often have to face fear and stigma related to the disease, complications of treatment. It is an opportunity for patients to help adjust to a “new normal” even though they have ongoing and emerging health problems. 26
2. Developing strategies and practices for sexuality care
The need to design and test known implementation strategies to integrate and disseminate scientific evidence-based interventions into practice for the benefit of cancer survivorship research. And the development of interventions such as implanting program in a gynecological oncology outpatient clinic formed by health professionals to receive information about sexual health care, the changes that may occur in their bodies and mainly an individual approach to realize the needs of each of the patients. 41 44
3. Individualized care plan
A care plan should be made based on an assessment of the needs of the patients and reach those who need rehabilitation to obtain long-term quality of life of their sexual function. 10 27 37 58 59 This individualized approach would also allow for greater flexibility and effective sexual health assessment with the possibility of discussing symptom management and strategies to improve sexual function and satisfaction. 41
4. Multiprofessional team support
Multiprofessional team support could help the sexual functioning of gynecological cancer survivors in the long term, as it is composed of physicians, psychologists, social workers, and physiotherapists. 43 Many women suffer with sequelae from treatment, such as vaginal discomfort, and physiotherapy treatment would help with pelvic floor exercises that could decrease this problem. The patients would also benefit from the help of psychologists accompanying them in this difficult period of changes, especially bodily and mental ones. 14 23 50 51
5. Development of rehabilitation programs
The development of rehabilitation programs for survivors of gynecological cancer is very important because they can help analyzing the discomforts of sexual complaints and improve health as a whole. 9 Many female cancer survivors receive part of the medical treatment; however, frequently, psychosocial issues are not properly addressed. This is problematic because psychosocial needs impact the lives of patients in various ways, both personal, social, psychological, and financial. However, existing survival care is often targeted with a focus on the disease, not covering other aspects of the life of the patient, such as the social stigma on cancer, which is often seen by society as a death sentence and with little chance of the patient having quality of life. However, there are experiences in studies that confirm that, with the implantation of intervention groups formed by professionals such as physiotherapists, sexual counselors, and psychologists helped patients in their sexual difficulties and resulted in improvements in their psychological and physical aspects. 23
6. Support networks
The creation of support networks aims to understand the emotional concerns and physical changes that interfere with the sexual identity of female cancer survivors and to offer the most appropriate support according to their specific needs. 42 Survivors have been living with or beyond cancer for a longer time, and there is a growing need to focus on health strategies that improve care and support to ensure a healthy and active life for as long as possible. 50 Because many women do not have their needs met, such as psychosocial and psychosexual needs, support networks corroborate so that they are welcomed, listened to, and supported to maintain their recovery and better manage the consequences of treatment. 9 40 44 Chart 2 demonstrates the supportive care strategies that can be offered to female cancer survivors post gynecological cancer treatment for sexuality complaints.

**Chart 2:** Matrix of healthcare strategies for post-treatment sexuality complaints

Study	Care Strategies					
	Patient-clinician communication	Development of rehabilitation programs	Multiprofessional team support	Individualized care plan	Development of strategies and practices for sexuality care	Creating a support network for survivors
Carter et al. (2010) 46	✓					
Levin et al. (2010) 47	✓					
Walton et al. (2010) 36		✓	✓			
Beesley et al.(2013) 54	✓			✓		
Grover et al. (2012) 37				✓		
Stavraka et al. (2012) 52				✓		
Le Borgne et al. (2013) 55			✓			
Afiyanti et al. (2013) 48	✓					
Sekse et al. (2013) 56	✓					
Loyd et al. (2014) 40	✓					✓
McCallum et al. (2014) 41	✓			✓	✓	
Vermeer et al. (2015) 18	✓			✓		
Hopkins et al. (2015) 49			✓			
Lee et al. (2015) 13	✓					
Rowlands et al. (2015) 42				✓		✓
Westin et al. (2015) 58				✓		
Bakker et al. (2016) 44		✓			✓	
Corrêa et al. (2016) 45	✓		✓			
Mikkelsen et al. (2016) 10				✓		
Bakker et al. (2017) 9		✓				
McCallum et al. (2017) 16						
Lutgendorf et al. (2017) 43			✓			
Teng et al. (2014) 57				✓		✓
Chow et al. (2018) 26	✓					
Mattsson et al. (2018) 50			✓			✓
Plotti et al. (2018) 27				✓		
Abbott-Anderson et al. (2020) 15	✓					
Fischer et al. (2019) 17	✓				✓	
Hubbs et al. (2019) 30	✓					
Bacalhau et al. (2020) 53						
Haryani et al. (2020) 51			✓			
Roberts et al. (2020) 23		✓				

## Discussion

The main objective of the present systematic review was to explore the main complaints in the field of sexuality with women survivors of cancer of the gynecological tract after treatment and to identify the care strategies that can be offered for these complaints.

When doing oncological treatment of the female tract, many negative symptoms occur in women, and each type of tumor causes an effect, such as vulvar cancer. Although surgery and treatment have become less radical over the decades, it can still cause scarring and mutilation of the external genitalia, such as local excision to radical vulvectomy and removal of the clitoris, which can affect various nerves and blood vessels involved in important sexual, anal, and/or urinary functions. 26 31 Thus, the main complaints of affected women resulting from this treatment is the high risk of psychological distress, low libido, and dissatisfaction in the relationship with their partner. 60 61 And because vulvar cancer is a neoplasm with a rare condition, there is scarcity of studies on the impact of the disease and little is known about the real emotional, social, and psychological impacts on surviving patients. 2 4 8 Studies suggest that, for these cases, the care strategy that can be offered to surviving patients is psychological and therapeutic support, because many of them have significant levels of suffering, with an altered perception of their body image and feelings of isolation and embarrassment resulting from external genital mutilation. 11 12 13

Patients who underwent treatment for cervical cancer may also have a worsening quality of life and complaints in the field of sexuality, because certain treatments, such as radiotherapy, induce long-term toxicity with decreased urinary and gastrointestinal function; as a consequence, the main complaints in sexuality is vaginal shortening and atrophy, leading to dyspareunia and loss of sexual desire. 13 As a care strategy to prevent the atrophy process that affects sexual function, early initiation of local estrogen therapy is recommended; however, unfortunately, there is no definitive therapeutic option that fully preserves the quality of life of cervical cancer patients, because each treatment induces more or less low self-esteem and variations in reactions to the treatment. 26 31

In the biopsychosocial aspect, a serious problem to be faced arising from the treatment performed for cervical cancer is stigma and discrimination; as found in a study conducted in Brazil, the respondents reported that they suffered prejudice and were considered by the society in which they lived as dirty, lazy, and promiscuous, and due to this condition, many of them had their social life impaired. 62

The studies reviewed highlighted that ovarian cancer is usually diagnosed late and that the disease is detected in advanced stages; however, thanks to recent advances in treatment options, the number of surviving women is increasing. 29 42 However, patients who underwent chemotherapy, radiation, or surgery to remove the ovaries experience as a complaint in sexuality the psychosexual morbidity (physical and psychological sexual problems), vaginal atrophy due to the drop in estrogen levels, and reduction of libido as a consequence of the reduction in testosterone and androstenedione levels. 35 43

Throughout the trajectory of the patient, cancer-related symptoms and side effects of the treatment cause significant psychosocial morbidity that directly reflects on their lives. 23 The psychosocial suffering related to sexual dysfunction and visual effects such as scars and hair loss may directly affect the perception of the own self-image of the woman, therefore, referral to specialists is necessary to treat this multiplicity of chronic problems that occur after treatment. 9 21 58 In the literature, it is widely reported that regular and prolonged use of vaginal dilators or vibrators can prevent and delay the development of vaginal adhesions and stenosis; the use of water-based lubricants and estrogen application are also recommended to promote vaginal regeneration and decrease sexual complaints; however, there are cases of women whose sexual health problems have become chronic, needing additional support. 35

In this sense, the strategy of doctor-patient communication regarding sexual health is essential and can help patients face these challenges. 23 However, this care strategy can be challenging in the oncologic scenario, since despite its importance, there are obstacles in this bond, and on several occasions, health professionals find it difficult to approach the sexuality and intimacy of the patient due to lack of time and training and to the fear that the patient will be embarrassed to talk about her personal life. 12 45

An intimate relationship is a psychosocial process strongly experienced in the female sexual function and many difficulties faced by women survivors are a result of this. 19 34 The partner perceives the changes that occur after treatment, whether physical or emotional, and in some cases the roles may change, with the partner being the main caregiver of the patient. 15 19 In the biopsychosocial aspect, this has a very relevant factor, because many of them feel unprepared and without the correct information of care, which brings a negative psychological, social, and even financial burden, because in many situations it is necessary to give up the job to be able to give the support that the patient needs. 11 13 60 Intimate intercourse is a psychosocial process strongly experienced in the female sexual function and many difficulties faced by surviving women are a result of this. 19 34 In addition, sexual dysfunctions are closely linked to the stages of the cycle of sexual response, that is, the inability of the sexual act to be satisfactory for the couple. And with women who have undergone cancer treatment, this is more evident because many lose their will and libido, not only because of their physical appearance, but also because they are very psychologically shaken, which further distances them from their partners.

The limitation of the present research lies in the design of the strategies used and are not considered to be other adverse symptoms that survivors face in the context of sexuality after gynecological treatment.

## Conclusion

The main complaints identified in the scientific literature regarding the post-treatment of cancer survivors were low libido and lack of interest in sexual activity. The care strategy that could be adopted would involve initial consultations with medical personnel and a thorough review of complaints. and symptoms reported patient's hair to find cause, which may be physical or psychological. Another complaint often referred to in the selected studies is vaginal dryness and, as a treatment strategy, the use of vagina moisturizers could improve humidification and thus reduce this discomfort in women. Pain during intercourse and stenosis or shortening of the vagina were also identified as common symptoms in women after cancer treatment; therefore, adopting healthcare strategies and actions can reduce pain and better deal with these complaints, such as mobilizing rehabilitation focused on sexual health with the participation of specialist professionals such as physiotherapists to analyze the case of each patient to help her in the best possible way. And, finally, sexual anxiety is another common complaint cited in the academic literature that causes severe mental and psychological problems for patients. An approach to care that could be used would be open communication and sincere dialogue between the physician and the patient with an integrative therapeutic approach to identify the major problems affecting them to provide an individualized care plan with the support of a multidisciplinary health team. It is suggested that future research should conduct qualitative studies to explore strategies that are acceptable in the perceptions of patients of suggesting improvements.
