# Supplementary material for: Management Strategies for Sexuality Complaints after Gynecologic Cancer: A Systematic Review
Source: Rev Bras Ginecol Obstet. 2022 Sep 29;44(10):962–71. doi: 10.1055/s-0042-1756312 (PMC9708405; doi:10.1055/s-0042-1756312)
Supplement: Supplementary file 1 — Supplementary Material [file 10-1055-s-0042-1756312-s210393.pdf]

**Appendix 1** Summary of the articles selected for the systematic review

| Author, Year | Country/region | Type of study | Participants | Type of cancer* | Phase of care continuum | Quality evaluation | | |
|---|---|---|---|---|---|---|---|---|
| | | | | | | Positive point | Negative point | Score |
| Carter et al. (2010)[46] | USA | Cross research | Sample size: 88<br>Age: 21 to 49 years old<br>Average age:<br>36 years old | Gynecological cancer | Postprimary treatment/<br>early survivorship | Highlights the long-term survivorship of women with advanced stage ovarian cancer and related. | Participation of healthier survivors in the survey may underrepresent the level of commitment of long-term survivors. | 3 |
| Levin et al. (2010)[47] | USA | Cross-sectional study and structured personal interviews | Sample size: 186<br>Age): 20–85 years oldAverage age:<br>54 years old | Gynecological cancer | Mixed phase care | Presents an association between sexual morbidity and adverse psychological. | Women with aggressive, rapidly progressing cancer or with significant medical morbidity were not included in the research results. | 3 |
| Walton et al. (2010)[36] | New Zealand | Unstructured interviews - a qualitative study | Sample size:<br>28Average age (years old): 50<br>Age range: 25 to 79 years old | Gynecological cancer | Postprimary treatment/<br>early survivorship | The issues of continuity and coordination of care that can provide supportive care after the end of treatment were discussed. | The participants in this study were from a healthcare facility in an isolated region. | 4 |
| Beesley et al.(2013)[54] | Australia | Longitudinal study | Sample size:<br>219Age: (29 to 85 years old<br>Average age:<br>59 years old | Cervical cancer | Postprimary treatment/<br>early survivorship | Women with ovarian cancer reported a need for continued assistance in the 1st 2 years after treatment. | Women with other types of gynecological cancer could be prioritized to better target unmet needs. | 4 |
| Grover et al. (2012)[37] | USA | Cross research | Sample size: 334<br>Average age:<br>49 years old<br>Age range: 18 to 83 years old | Gynecological cancer | Postprimary treatment/<br>early survivorship | Patient-reported side effects were reported, especially concerning sexual dysfunction. | Other patient-focused tools to evaluate these collateral effects and access to survivorship. | 3 |
| Stavraka et al. (2012)[52] | United Kingdom | Cross study | Sample size:<br>116Age range: 31 to 88 years old<br>Average age:<br>62 years old | Cervical cancer | Postprimary treatment/<br>early survivorship | Patients after treatment for ovarian cancer were described and compared with symptoms documented in their hospital records. | There was a significant disagreement between symptoms reported in the questionnaire and those recorded in hospital records. | 3 |
| Afiyanti et al. (2013)[48] | Indonesia | Descriptive research and in-depth interviews | Sample size: 13<br>Average age:<br>40 years old<br>Age range: 38 to 48 years old | Cervical cancer | Postprimary treatment/<br>early survivorship | Understanding of how women adapt to their altered sexuality and intimacy with their partner after cancer treatment. | The small sample size and the participants in this study may not have expressed their true feelings when discussing their intimacy experiences and preferences. | 3 |
| Harter et al. (2013)[14] | Germany | Cross research<br>Focus group | Sample size: 727<br>Age range: 18 to 86 years old<br>Average age:<br>62 years old | Gynecological cancer | Mixed-phase care | Sexual function and activity in patients with gynecological cancer were investigated in a control group. | The response rate was low, and it was not possible to capture changes in priorities between the cancer survivor groups. | 4 |
| Le Borgne et al. (2013)[55] | France | Cross-sectional study (focus group) | Sample size: 173<br>Age range: 44 to 65 years old | Cervical cancer | Long-term survivorship | The study revealed data that even 15 years after the end of gynecological cancer treatment.. | A cross-sectional study impedes any evaluation of change in the quality of life in the group of selected survivors. | 3 |

(*Continued*)

 © 2022. Federação Brasileira de Ginecologia e Obstetrícia. All rights reserved.

**Appendix 1** (*Continued*)

| Author, Year | Country/region | Type of study | Participants | Type of cancer* | Phase of care continuum | Quality evaluation | | |
|---|---|---|---|---|---|---|---|---|
| | | | | | | Positive point | Negative point | Score |
| Sekse et al. (2013)[56] | Norway | Phenomenological, descritptive, interpretative approach with in-depth interviews | Sample size: 16 Age range: 39 to 66 years old Average age: 56 years old | Gynecological cancer | Long-term survivorship | Experiences the altered female body after gynecological cancer 5 to 6 years after treatment. | The experiences of gynecological cancer survivors could have been included with other ethnic groups and cultural backgrounds. | 3 |
| Loyd et al. (2014)[40] | United Kingdom | Descriptive and qualitative phenomenological study with semi-structured interviews | Sample size: 12Average age: 29 to 45 years old | Cervical cancer | Mixed-phase care | Overview of the experiences of women years after a trachelectomy identification for prevention of long-term physical effects. | Due to an increasing number of cancer survivors, it would be important to identify women at further stages. | 3 |
| McCallum et al. (2014)[41] | Canada | Descriptive, cross-sectional study with application of questionnaires. | Sample size: 113Age range: 27 to 89years old. Average age: 61 years old | Gynecological cancer | Mixed phase care | Women were followed and highlighted several supportive care sexual health needs. | The perceptions captured in the survey may have been personal and this implies a subjective experience report of a need. | 4 |
| Teng et al. (2014)[57] | Canada | Cross-sectional research (cross-sectional study) | Sample size: 102Age: 29 to 85 years old Average age: 58 years old | Cervical cancer | Mixed-phase care | Data on quality of life concerns and key patient needs were represented to establish priorities for future interventions. | Different stages of treatment and remission, which may underestimate the true effects on quality of life with a uniform patient population. | 3 |
| Vermeer et al. (2015)[18] | Holland | Multicenter cross-sectional study | Sample size: 343 Age: -Average age: 50 years old | Cervical cancer | Postprimary treatment/ early survivorship | Prevalence of psychosexual health needs and help-seeking behavior among gynecological cancer survivors. | The study design only permitted the collection of retrospective data, making it impossible to make causal inferences. | 3 |
| Hopkins et al. (2015)[49] | United Kingdom | Cross-sectional research (mixed method) | Sample size: 102Age range: 18 to 77 years old Average age: 51 years old | Ovary cancer | Postprimary treatment/ early survivorship | Symptoms of sexual functioning and persistent psychological and physical problems were clearly described by participants. | Limited analysis of associations between treatment and sexual dysfunction may have occurred. | 4 |
| Lee et al. (2015)[13] | Taiwan | Semistructured interviews - a qualitative study | Sample size: 11Average age: 47 years old Age range: 20 to 60 years old | Gynecological cancer | Postprimary treatment/ early survivorship | Sexual experiences in the sociocultural context of Chinese women after gynecological cancer were reported. | Generalization of the results to women of other age groups and the small sample could prevent an investigation of a wide range of other cancers. | 3 |
| Rowlands et al. (2015)[42] | Australia | Unstructured interviews - a qualitative study | Sample size: 237Average age: 65 years old | Endometrial cancer | Long-term survivorship | Long-term changes experienced by women treated for endometrial cancer were examined. | Quantitative measures may not capture all worries and changes of the women studied. | 4 |
| Vermeer et al. (2016)[59] | Holland | A qualitative study with semistructured interviews | Sample size: 30 Average age: 47 years old | Gynecological cancer | Mixed-phase care | Sexuality was found to be a taboo topic and, therefore, made it difficult for some participants to seek help, despite wanting information about the consequences of treatment for sexual | It was reported that, during the course of the research, some participants found it difficult to report their own experiences of sexual dysfunction and psychosexual support. | 4 |

 © 2022. Federação Brasileira de Ginecologia e Obstetrícia. All rights reserved.

**Appendix 1** (*Continued*)

| Author, Year | Country/region | Type of study | Participants | Type of cancer* | Phase of care continuum | Quality evaluation | | |
|---|---|---|---|---|---|---|---|---|
| | | | | | | Positive point | Negative point | Score |
| | | | | | | functioning and practical advice on how to cope with dysfunction. | | |
| Westin et al. (2015)[58] | USA | Prospective study | Sample size: 1,029Average age (years): 59 years old | Gynecological cancer | Postprimary treatment/ early survivorship | The main health-related problems faced by gynecological cancer survivors. | No validated instruments were used to conduct the research. | 3 |
| Bakker et al. (2016)[44] | Holland | Cross study | Sample size: 194Age range: 25 to 69 years old | Cervical cancer | Postprimary treatment/ early survivorship | Assessment of sexual distress among cervical cancer survivors and association with sexual symptoms. | There were no measurements that could be compared with those of a group of women with no history of gynecological cancer. | 2 |
| Corrêa et al. (2016)[45] | Brazil | Prospective research (8 years of follow-up) | Sample size: 74Average age: 51 years old Age range: 24 to 85 years old | Gynecological cancer | Mixed-phase care | It was demonstrated how treatment can have a negative impact on the sexual function of women. | Many out-of-date medical records and abandonment of screening for the disease were found. | 4 |
| Mikkelsen et al. (2016)[10] | Denmark | Cross study | Sample size: 85Age: 45 and 65 years old | Cervical cancer | Mixed phase care | Cervical cancer survivors who received chemotherapy had a serious risk of developing late adverse effects and greater rehabilitation needs. | The sample size was small, and all information was self-reported by the participants themselves, which can be a problem. | 3 |
| Bakker et al. (2017)[9] | Holland | Cross-sectional study and semistructured interviews | Sample size: 20Average age: 40 years old Age range: 26 to 71 years old. | Gynecological cancer | Mixed-phase care | A nurse-led sexual rehabilitation intervention aimed at sexual recovery and vaginal dilatation. | Lacked consistent evidence for the association of reduced vaginal complaints and sexual rehabilitation. | 3 |
| Lutgendorf et al. (2017)[43] | USA | Cross research | Sample size: 56Age range: 40 to 85 years old Average age: 65 years old | Ovary cancer | Long-term survivorship | The long-term survival of women with ovarian cancer was investigated. | Some people approached at the study sites declined due to poor health, and with this, it is not possible to know if the healthier survivors. | 3 |
| McCallum et al. (2017)[16] | Canada | Cross research | Sample size: 113Age: 27 to 89 years oldAverage age: 60 years old | Gynecological cancer | Postprimary treatment/ early survivorship | Several unmet post-treatment needs were studied especially for younger survivors. | It was not possible to screen the number of potential patients to calculate an accurate recruitment rate. | 4 |
| Chow et al. (2018)[26] | China | Cross study | Sample size: 225 Average age: 32 years old Age range: 21 to 60 years old | Gynecological cancer | Postprimary treatment/ early survivorship | It has reported the various challenges for survivors in the process of adjustment to the disease. | Diagnosis of gynecological cancer was not assessed so that changes in such outcomes could be compared. | 2 |

(*Continued*)

 © 2022. Federação Brasileira de Ginecologia e Obstetrícia. All rights reserved.

**Appendix 1** (*Continued*)

| Author, Year | Country/region | Type of study | Participants | Type of cancer* | Phase of care continuum | Quality evaluation | | |
|---|---|---|---|---|---|---|---|---|
| | | | | | | Positive point | Negative point | Score |
| Mattsson et al. (2018)[50] | Sweden | Survey questionnaire | Sample size: 337Average age: 32 years old Age range: 19 to 39 years old | Gynecological cancer | Postprimary treatment/ early survivorship | Investigates a set of variables; in addition, potential participants were identified with gynecological cancer. | It was not possible to detect clinically relevant levels of distress to answer the study objective, both in terms of breadth and depth. | 4 |
| Plotti et al. (2018)[27] | Italy | Retrospective protocol study (10 years of follow-up) | Sample size: 251Average age: 55 years old | Cervical cancer | Postprimary treatment/ early survivorship | Assesses the long-term impact on quality of life, urinary dysfunction, and sexual function of patients affected by cervical cancer. | The patients selected for the study were those exclusively treated at the Campus Bio-Medico in Rome, excluding other institutions. | 3 |
| Abbott-Anderson et al. (2020)[15] | USA | Cross-sectional research + focus groups | Sample size: 261 Average age: 60 years old | Gynecological cancer | Mixed-phase care | Identified several factors that could improve sexual well-being, and thus provide opportunities for interventions with couples. | Some participants reported personal beliefs which inevitably may not express an understanding of other participants with other cultures and beliefs. | 3 |
| Fischer et al. (2019)[17] | Canada | Cross-sectional descriptive study and focus group | Sample size: 64Age range: 51 to 64 years oldAverage age: 54 years old | Ovary cancer | Postprimary treatment/ early survivorship | Sexuality in quality of life and the impact ovarian cancer can have on the sexual life of women. | May be limited to conclusions and comparisons of endpoints of interest. | 3 |
| Hubbs et al. (2019)[30] | USA | Cross-sectional study | Sample size: 85Average age: 52 years old | Gynecological cancer | Mixed phase care | Sexual dysfunction in gynecological cancer patients and highlights the challenges that healthcare providers and patients. | May not reflect changes in perceived sexual function at different time points after treatment. | 4 |
| Bacalhau et al. (2020)[53] | Portugal | Cross study | Sample size: 113 women Age range: 23 to 67 years old Average age: 48 years old | Cervical cancer | Postprimary treatment/ early survivorship | The importance of psychoeducational programs to help patients cope with disturbing sexual and vaginal functioning. | If the research adopted the longitudinal design, it could provide more information on sexual satisfaction after treatment. | 3 |
| Haryani et al. (2020)[51] | Indonesia | Cross-sectional study | Sample: 298 Average age: 50 years old | Gynecological cancer | Postprimary treatment/ early survivorship | Clinical benefits in assessing supportive care needs among gynecological cancer patients. | It would be necessary to evaluate this tool in other cancer patient populations and thus produce significant correlations. | 4 |
| Roberts et al. (2020)[23] | Canada | Prospective study - control group | Sample size: 78Age range: 29 to 80 years old Average age: 56 years old | Gynecological cancer | Postprimary treatment/ early survivorship | Screening questionnaire in the outpatient clinic identified sexual health concerns in more than half of gynecological cancer survivors. | There were wide differences between the control groups in the study, which could distort the results obtained. | 4 |

Note: Gynecological cancer* includes all types of neoplasms of the female genital tract: cervix, vagina, ovarian, and endometrium.

 © 2022. Federação Brasileira de Ginecologia e Obstetrícia. All rights reserved.
